# New-onset cutaneous lichen planus following Vaxzevria (Oxford-AstraZeneca) COVID-19 vaccination

**DOI:** 10.1177/2050313X241307109

**Published:** 2024-12-20

**Authors:** Amira Muftah, Stephen Lee, Mariam Abbas

**Affiliations:** 1 Department of Dermatology, College of Medicine, University of Calgary, Calgary, AB, Canada; 2 Department of Infectious Diseases, College of Medicine, University of Saskatchewan, Regina, SK, Canada; 3 Department of Dermatology, College of Medicine, University of Saskatchewan, Regina, SK, Canada

**Keywords:** Dermatology, inflammatory dermatoses, infectious disease

## Abstract

We report a 56-year-old male who developed cutaneous lichen planus (LP) following Vaxzevria (Oxford-AstraZeneca) COVID-19 vaccination. Multiple topical and systemic therapies were tried with limited success; however, partial improvement was observed with narrow-band UVB (NB-UVB) phototherapy. This report adds to the growing evidence of new-onset LP following COVID-19 vaccination and underscores the need for careful reporting, monitoring, and management of vaccine-related adverse effects.

## Introduction

Lichen planus (LP) is an inflammatory disease affecting the skin, nails, hair, and/or mucosae.^1–4^ The clinical presentation of LP varies depending on the site of involvement. Cutaneous LP classically presents as pruritic, polygonal, flat-topped, violaceous papules and plaques with characteristic Wickham striae, reticulated white or gray overlying scales.^1–4^ LP may be further categorized by morphological subtypes including papular (classic), linear, annular, follicular, hypertrophic, atrophic, vesicobullous, erosive, actinic, palmoplantar, pigmentosus, pigmentosus-inversus, and lichen planopilaris.^1–4^ Skin biopsy is the definitive diagnostic test.^1–4^

The pathogenesis of LP is not fully understood; however, genetic and immunologic factors are implicated. In general, activated CD8+ (cytotoxic) T lymphocytes and CD4+ (helper) T lymphocytes stimulate the recruitment of cytokines and other immune mediators including interferon-γ (IFN-γ) and tumor necrosis factor-α (TNF-α) which induce basal keratinocyte apoptosis.^1–4^ This autoimmune response is thought to be triggered by various environmental factors, including vaccines, infections, medications, dental materials, tobacco use, and stress.^1–7^

The ongoing development and dissemination of COVID-19 vaccines have raised concerns about their potential adverse effects. Several studies have reported new-onset or worsening LP following vaccination with Tozinameran (Pfizer-BioNTech), Spikevax (Moderna), Vaxzevria (Oxford-AstraZeneca), Jcovden (Johnson and Johnson), and others.^8–19^ The pathogenesis underlying the association between COVID-19 vaccines and LP remains unclear. We report a case of new-onset LP following a first dose of the Vaxzevria vaccine to further inform the growing body of evidence on COVID-19 vaccine-induced LP.

## Case report

A 56-year-old male with a history of hypertension, dyslipidemia, osteoarthritis, and degenerative disk disease presented to our dermatology clinic with an 18-month history of non-resolving, pruritic, erythematous to violaceous papules and plaques. The rash initially appeared on his left calf 8–10 days following his first dose of the Vaxzevria COVID-19 vaccine in April 2021.

His medications included hydromorphone, gabapentin, rosuvastatin, losartan potassium-hydrochlorothiazide, and esomeprazole. There was no personal or family history of dermatologic disease. He denied potential triggers including infections, surgeries, medication adjustments, dental interventions, psychosocial stressors, or other vaccinations. He had a remote smoking history of 5-pack-years.

He initially presented to his family physician 10 days following the onset of the rash and was prescribed Ketoderm 2% cream with no improvement. A punch biopsy of the rash was subsequently performed and revealed hyperkeratosis and hypergranulosis with lichenoid inflammatory infiltrate in the superficial dermis in addition to basal epidermal disruption with numerous Civatte bodies and pigmentary incontinence. There was no evidence of vasculitis, eosinophils, or granulomatous inflammation.

In view of the clinical history and histopathological findings, the patient was diagnosed with vaccine-induced LP and concurrently managed by his family physician and a dermatologist. Prior to his presentation to our clinic, he was treated with betaderm 0.05% ointment, tacrolimus 0.1% ointment, calcipotriene 0.005% and betamethasone dipropionate 0.064% foam, acitretin, metronidazole, naltrexone, and prednisone with refractory improvement. Notably, subsequent doses of the Tozinameran (Pfizer-BioNTech) COVID-19 vaccine did not exacerbate his symptoms. Given the patient’s recalcitrant course, he was referred to our dermatology clinic for further evaluation and management.

**Figure 1.**
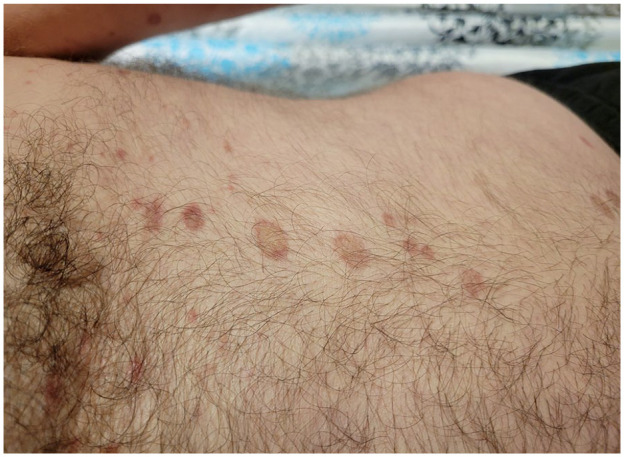
Erythematous, annular papules and plaques with central clearing and overlying fine scale on the trunk.

**Figure 2.**
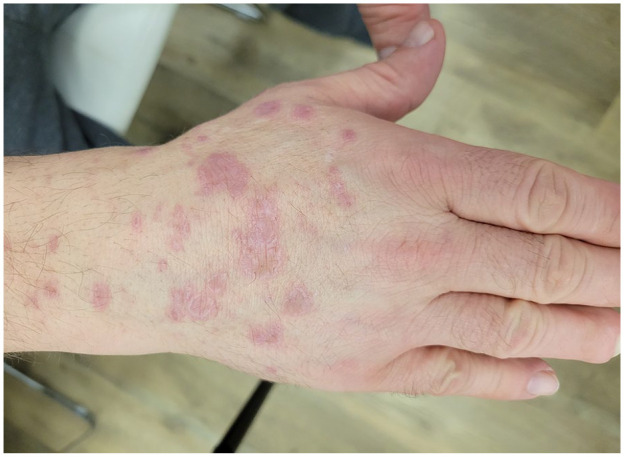
Erythematous to violaceous annular papules and plaques with raised borders and overlying fine scale on the dorsal right hand and wrist.

**Figure 3.**
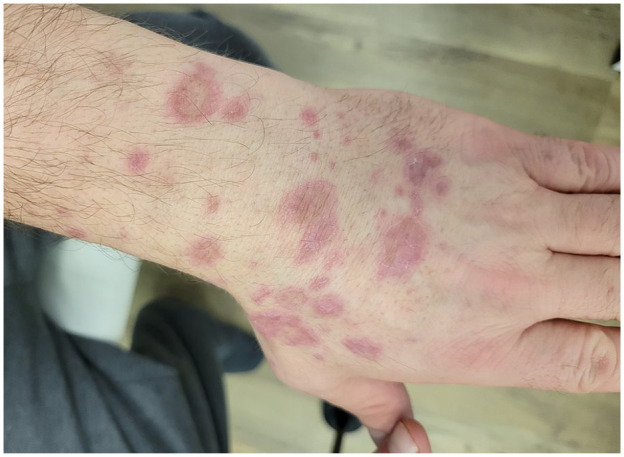
Erythematous to violaceous annular papules and plaques with raised borders and overlying fine scale on the dorsal left hand and wrist.

**Figure 4.**
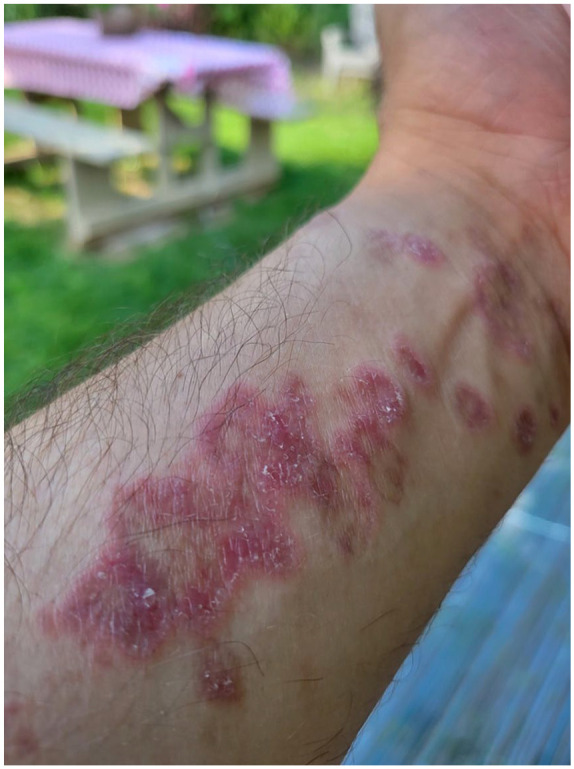
Erythematous to violaceous, annular, coalescing papules and plaques with raised borders and overlying fine scale on the volar left forearm and wrist.

**Figure 5.**
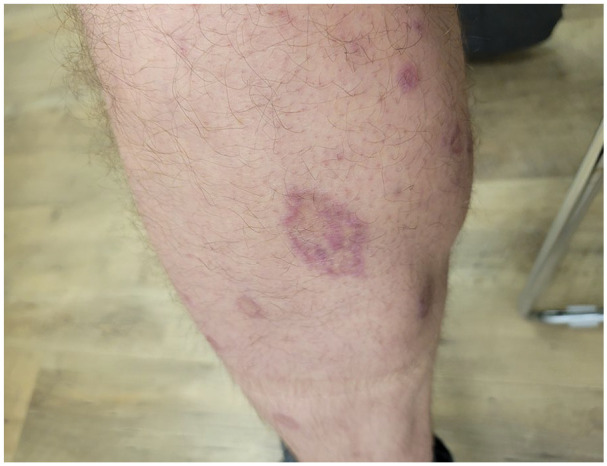
Violaceous annular papules and plaques with central clearing and overlying fine scale on the left calf.

At presentation to our clinic, physical examination (Figures 1–5) revealed well-demarcated, erythematous to violaceous annular papules and plaques with fine scale on the dorsal hands and wrists, forearms, dorsal feet, calves, and trunk with a total body surface area of less than 15%. He had no nail changes, mucosal lesions, or systemic symptoms.

Further investigations revealed a positive anti-nuclear antibody at a titer of 1:160 and an anti-histone antibody. Two additional punch biopsies of the rash on the right forearm were performed and demonstrated consistent histologic features including a lichenoid inflammatory infiltrate obscuring the dermal-epidermal junction with numerous colloid bodies and lymphocytic exocytosis. The epidermis showed wedge-shaped hypergranulosis and orthokeratosis. The inflammatory infiltrate was composed of lymphocytes, histiocytes, and scattered melanophages. Eosinophils and fungal elements were not appreciated.

**Figure 6.**
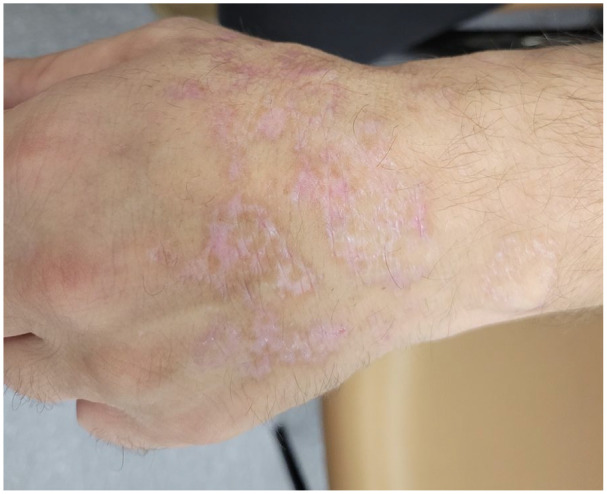
Post-inflammatory hypo- and hyper-pigmentation on the dorsal left hand following NB-UVB phototherapy.

**Figure 7.**
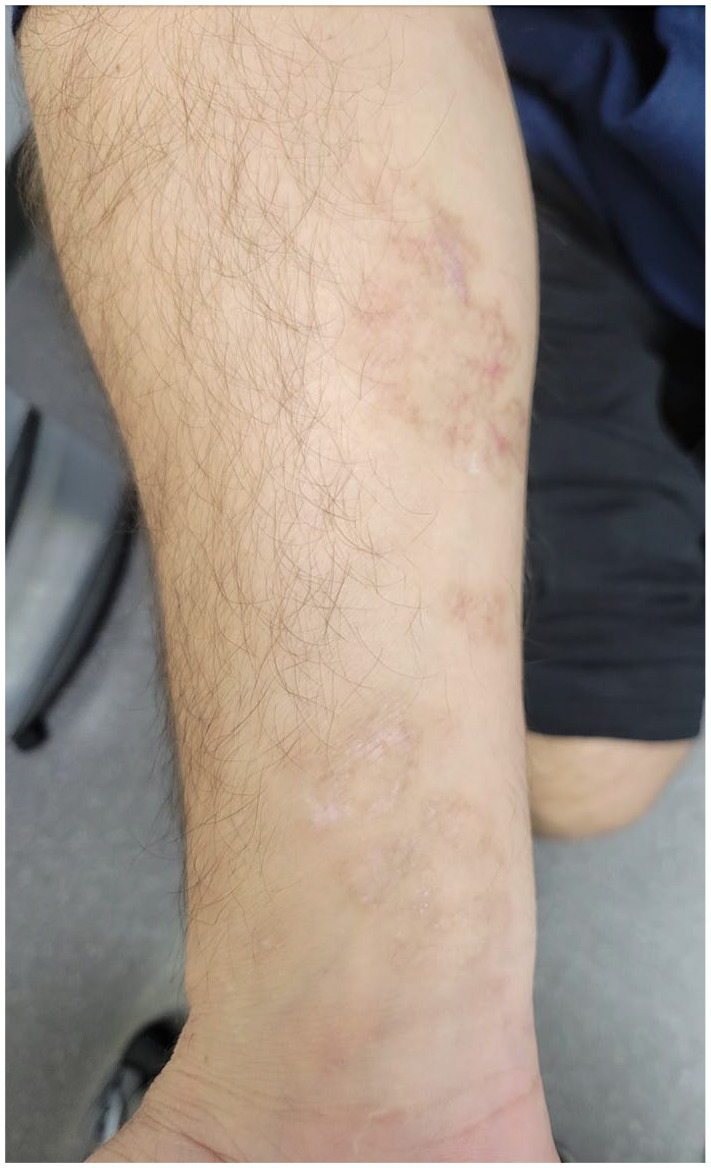
Post-inflammatory hypo- and hyper-pigmentation on the volar right forearm following NB-UVB phototherapy.

**Figure 8.**
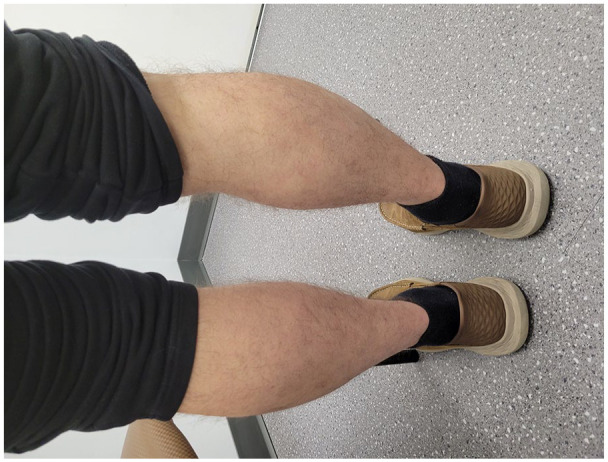
Clearance of skin on the bilateral calves following NB-UVB phototherapy.

Given the chronological sequence of the rash relative to the administration of the Vaxzevria vaccine, the absence of other triggers, and histopathological findings, the diagnosis of vaccine-induced LP was reaffirmed. In view of his recalcitrant course to numerous topical and systemic therapies, hydroxychloroquine was initiated but discontinued due to gastrointestinal effects. Topical roflumilast was also tried with minimal improvement. He has recently commenced NB-UVB phototherapy, which has led to significant improvement in his skin and pruritus (Figures 6–8).

## Discussion

This case report describes a 56-year-old male who developed new-onset, biopsy-proven, cutaneous LP 8–10 days after receiving the first dose of the Vaxzevria COVID-19 vaccine. LP is a chronic, inflammatory disease that affects the skin, hair, nails, and/or mucosae.^1–4^ While the pathogenesis of LP is not fully understood, it represents a T-cell-mediated autoimmune response.^1–4^ This response is triggered by several immunogenetic, environmental, and clinical factors. There is evidence to suggest that systemic diseases (e.g., diabetes, hypertension, dyslipidemia, thyroid disease, chronic liver disease, and malignancy), infections (e.g., hepatitis C, hepatitis B, human papillomavirus, and herpes simplex virus), dental amalgam, tobacco use, and stress increase susceptibility to LP.^1–7^ Given this, the patient’s longstanding history of hypertension, dyslipidemia, and remote smoking history may have influenced his susceptibility to LP. Certain medications, such as diuretics, beta-blockers, ACE inhibitors, antimalarials, and NSAIDs are associated with drug-induced LP.^2,4,5,8^ Although the patient was on a diuretic at the onset of the rash, this was a longstanding medication and therefore, unlikely a provoking factor. Moreover, LP may be distinguished from lichenoid-drug eruptions due to the absence of eosinophils on histopathology, as seen in our case.^1,4,5^

The association between LP and vaccines, such as the hepatitis B vaccine, has been well-established.^20^ Several cases of new-onset and exacerbation of LP following COVID-19 vaccination have been reported.^8–19^ A review by Zou and colleagues identified 39 cases of new-onset or relapse of LP following various vaccines, including Tozinameran (Pfizer-BioNTech), Spikevax (Moderna), Jcovden (Johnson and Johnson), Vaxzevria, Sinopharm, and CoronaVac.^8^ Vaxzevria was implicated in 8 of these cases.^8^ Consistent with our case, the median latency from COVID-19 vaccination to the onset of LP was 9 days.^8^ Most cases demonstrated resolution or marked response within 3 months of therapy; however, our patient demonstrated a recalcitrant course, with only partial improvement with NB-UVB phototherapy.^8^

A prevailing hypothesis in the pathogenesis of COVID-19 vaccine-induced LP involves autoimmunity, whereby exposure to COVID-19 antigens via vaccination activates CD8+ (cytotoxic) T-cell lymphocyte response against basal keratinocytes in the epidermis leading to apoptosis and subsequent manifestation of LP.^2,8,9^ In addition, the activation of CD4+ (helper) T lymphocytes in the T-cell type 1 (Th1) response release proinflammatory cytokines, including IFN-γ and TNF-α, ultimately maintaining the Th1 response and promoting keratinocyte apoptosis.^2,8,9^

Although vaccine-induced LP is a rare adverse event, vaccination remains a critical tool in reducing COVID-19 morbidity and mortality. Further research is necessary to clarify the association between vaccinations, including those for COVID-19, and LP, as well as to understand potential factors contributing to the recalcitrant course of vaccine-induced LP.
